# Microsurgical treatment of tentorial meningiomas: Report of 30 patients

**DOI:** 10.4103/2152-7806.66851

**Published:** 2010-07-29

**Authors:** Paulo Henrique Aguiar, Adriana Tahara, Antonio Nogueira de Almeida, Kaoru Kurisu

**Affiliations:** Department of Neurology, São Paulo University Hospital, Dr Eneas de Carvalho Aguiar Avenue, 647 – Cerqueira César, 05403-900, São Paulo, Brazil; 1 Department of Neurosurgery, Hiroshima University Hospital, Kasumi 1-2-3, 734-8551, Hiroshima, Japan

**Keywords:** Brain tumor, morbidity, mortality, neurosurgery, tentorial meningioma

## Abstract

**Background:**

Tentorial meningiomas represent about 5% of intracranial meningiomas. This article reviews our recent institutional series of patients with tentorial meningiomas, proposes a simplified classification and analyzes postoperative evolution, discussing the salient features in the management of these patients.

**Methods:**

From 1998 to 2005, 30 patients (22 female and 8 male) with tentorial meningiomas were operated at our institution. Thirteen patients had tumor restricted to the infratentorial space; 12, to the supratentorial space; and in 5 cases, the tumor involved both compartments. Follow-up ranged from 1 to 8 years. A total of 35 surgical procedures were performed in 30 patients, where 26 procedures were performed through a single approach (2, ITSC; 10, RS; 5, SOIH; 5, ST; and 4, TT); and 9, through combined approaches (7, ITSC/ SOIH; and 2, RS/ST).

**Results:**

Simpson I resection was achieved in 17 patients. Tumors involving both compartments, involving the petrous sinus, and attached to the torcula limited complete resection. Twenty-two out of 30 patients were able to return to their regular life with no or minimal neurological sequelae. Most frequent complications in our series were shunt dependence, CSF fistulae, diffuse brain injury and visual field defects. Overall, our series revealed 3% mortality and 23% morbidity.

**Conclusion:**

Tentorial meningiomas are associated with significant morbidity related to the nervous and vascular structures surrounding the tumor. Partial tumor removal may be necessary in some cases.

## INTRODUCTION

Tentorial meningiomas are relatively uncommon tumors, representing about 5% of intracranial meningiomas reported in the literature.[1] Approximately 70% to 80% of cases occur in women.[11] Signs and symptoms of cranial hypertension are the most common findings, followed by cerebellar ataxia, psychiatric disturbances and cranial nerve dysfunction.[12] The tentorium seems to have a simple structural design; however, its edges are close to the brainstem.[21] Moreover, its role in venous drainage of the brain and cerebellum makes surgical approach a challenge, even for experienced neurosurgical teams.[18]

The first historic attempts at tentorial meningioma removal resulted in high rates of mortality and morbidity. In series published up to 1990, the mortality rate ranged from 14% to 44%.[3,6,17,27] Following the development of diagnostic imaging and neurosurgical techniques, mortality rates fell, reaching rates of around 10% in most series published over the last two decades.[28,32] Nonetheless, postoperative morbidity has continued to range from 18.9% to 77%.[2,5,8]

The objective of this paper was to review our institutional series of patients with tentorial meningiomas, analyze results and carry out postoperative clinical evaluation.

## MATERIALS AND METHODS

From 1998 to 2005, 30 patients (22 female and 8 male) with tentorial meningiomas were operated on at the University of Sao Paulo. Patients’ age ranged from 24 to 86 years. Patients having tumors located predominantly over the petrous bone or the clivus were excluded from this study. Histological specimens were obtained for all patients where diagnosis was based on the WHO classification.[26] Preoperative CT scan was obtained from 30 patients; MRI, from 28; and digital subtraction angiography (DSA), from 16 patients when Magnetic Ressonance Angiography (MRA) information was not satisfactory or when it was judged necessary (4 of these underwent preoperative arterial embolization). The 2 patients without preoperative MRI investigation arrived at the hospital with hydrocephalus in a poor neurological state and underwent urgent surgery.

MRI detected 6 patients with hydrocephalus, while DSA revealed total obstruction of the transverse sinus in another 2 patients. No aneurysm or arteriovenous malformations were found in our series.

**Figure 1:**
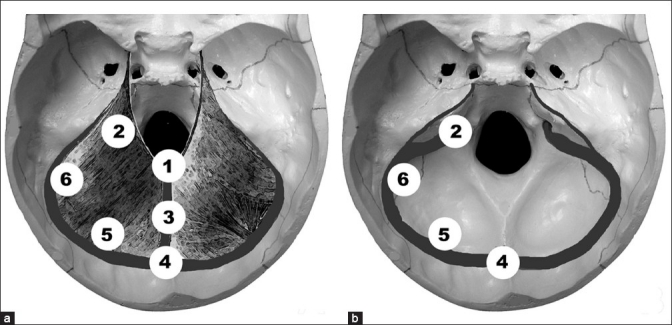
Schematic depiction of classification used in our series; (a) view from above the tentorium and (b) view of bone structures below the tentorium related to the tumor sites

On an average, tumors measured 4.3 cm (±1 cm) on their largest diameter [Table 1]. Thirteen patients had tumors limited to the infratentorial space; 12, to the supratentorial space; and in 5 cases, tumors involved both compartments. Tentorial tumors were divided into six groups according to their position. The classification is a modification of that proposed by Yasargil in 1996.[34] Group 1 comprised tumors related to the posteromedial free edge of the tentorium; group 2, tumors located at the anterolateral free edge of the tentorium; group 3, tumors between the torcula and the free edge of the tentorium; group 4, tumors attached predominantly to the torcula; group 5, tumors between the torcula and the transverse sinus; and group 6, tumors attached close to the petrous sinus. If the tumor involved more than one area, the group numbers corresponding to each area were summed. Letter ‘A’ was suffixed to the group number or the sum of group numbers if the lesion was supratentorial, letter ‘B’ if it was infratentorial, or ‘AB’ if the lesion involved both compartments (A comes first if the lesion was predominantly located in the supratentorial area, and B comes first if the lesion was predominantly infratentorial.) [Figure 1]. Classification of tumors of this series based on their size and location is presented in Table 1.

**Table 1:** Patients data showing classification, tumor size, pre and pos operative status, results and complications: resection is shown according to Simpson’s classification; follow-up period is in months; in the surgical approach, the / symbol indicates that approaches were performed at the same time, while the + sign indicates that approaches were performed on different days; size represents the largest diameter of the lesion in centimeters

ID	Age	Sex	TU class.	Preop hydrocephalus	Treat hydrocephalus	Resection	Follow-up (months)	Postoperative KPS	Surgical complications	Pathology	Adj. Ther.	Surg. Approach	Size (cm)	Recurrence (months)
1	47	F	6B	no	NA	II	84	100	no	benign	no	RS	2.5	no
2	52	F	6B	no	NA	I	30	100	no	atypical	RDT	RS	3.5	no
3	27	F	1AB	yes	EDV-VPS	I	40	100	Bleeding + small visual defect	benign	no	SOIH + ITSC/SOIH	4.5	no
4	58	F	4B	no	NA	II	18	80	no	atypical	RDT, RSG	ITSC/SOIH	5.5	no
5	24	F	5AB	no	NA	I	20	100	CSF fistula	benign	no	RS/ST	4	no
6	56	F	6A	no	NA	I	21	100	no	benign	no	TT	4.7	no
7	35	F	6B	no	NA	III	66	100	no	benign	RDT, RSG	RS	3	12
8	39	M	56A	no	NA	II/II/II	72*	80	CSF fistula	malignant	RDT	TT + TT + ST	4.5	72
9	68	F	4B	no	NA	I	NA	Death	TS ligated, CSF fistula, TEP, death	benign	no	ITSC/SOIH	5.5	NA
10	54	M	6A	no	NA	I	96	100	no	benign	no	ST	4.2	
11	62	M	6A	no	NA	I	75	100	no	benign	no	ST	5	60
12	65	F	6B	no	NA	I	32	100	CSF fistula	benign	no	RS	4	no
13	51	F	3A	no	NA	I	44	100	no	benign	no	SOIH	3.5	no
14	46	F	4B	yes	EDV-VPS	I	41	80	no	benign	no	ITSC/SOIH	4.5	no
15	75	F	6B	no	NA	I	47	100	no	benign	no	RS	5	no
16	37	F	3A	yes	EDV	I	14	100	no	benign	no	SOIH	5.4	no
17	56	F	6B	no	NA	I	55	100	no	benign	no	RS	4	no
18	33	F	5B	no	NA	II	23	50	severe brain edema	benign-NF2	RDT	RS	4	no
19	41	M	6AB	no	NA	II	63	100	TS ligated without deficit, CSF fistula	benign	no	RS/ST	5	no
20	66	M	5A	yes	EDV	I	96	80	no	benign	no	SOIH	6.5	no
21	62	M	6B	no	NA	II	94	100	no	benign	no	RS	3.7	no
22	62	F	4AB	yes	EDV	III	96*	80	hypovolemic shock, CSF fistula	benign***	RDT+ RSG	ITSC + ITSC/SOIH	6	90
23	32	F	2A	no	NA	II	41	90	no	benign	no	ST	1.8	no
24	54	F	6B	no	NA	II	72	100	no	benign	no	RS	4.2	no
25	32	F	6A	no	NA	I	84	100	no	benign	no	ST	3	no
26	42	M	12A	no	NA	IV/I	91	70	bleeding, brain swelling, Vis, CSF fistula	benign	no	ITSC + ITSC/SOIH	4.4	no
27	40	F	45AB	no	NA	II	25	100	TS ligated without deficit	benign	no	ITSC/SOIH	4.6	no
28	36	F	3A	no	NA	I	46	100	no	benign	no	SOIH	3	no
29	32	F	5A	no	NA	II	36	100	no	benign	no	TT	3.5	no
30	86	M	6B	yes	EDV	II	48**	80	no	benign	no	RS	4.6	NA

Abbreviations: ID- patient identity; TU class- tumor classification by site (see text for details); Adj. Ther.- adjuvant therapy; Preop - preoperative; Treat- treatment; EVD- external ventricular drainage; VPS- ventriculoperitoneal shunt; Surg. Approach- surgical approach;

* patients died due to progression of the disease;

** patient died due to a condition unrelated to the disease;

*** the tumor recurred and the second pathology disclosed a malignant transformation; NA- not applicable; Vis- patients developed postoperative visual impairment; TS- transverse sinus; NF2- patient had neurofibromatosis type 2; RS- retrosigmoid; SOIH- suboccipital interhemispheric; ITSC- infratentorial supracerebellar; TT- transtemporal; ST- subtemporal; RSG- radiosurgery; RDT- radiotherapy.

Surgical approaches were chosen taking into account position and venous involvement of tumors. Several surgical approaches were used separately or in combination in this series: a- infratentorial supracerebellar (ITSC) approach; b- retrosigmoid (RS); c- subtemporal (ST); d- suboccipital interhemispheric (SOIH); and e- transtemporal (TT) approach. A total of 26 procedures were performed through a single approach (2, ITSC; 10, RS; 5, SOIH; 5, ST; and 4, TT); and 9, through combined approaches (7, ITSC/ SOIH; and 2, RS/ST).

### Symptoms

Headache was the most common symptom in our series, being complained of by 75% of patients; followed by vertigo (50%), gait disturbance (45%), mental abnormality (41%), visual disturbance (38%), hearing loss (28%), seizures (28%), hemiparesis (19%), hemianesthesia (19%), tinnitus (19%) and facial pain (9%). Due to large volume of the tumors, we attribute the cause of the headache to intracranial hypertension, mainly in the patients with hydrocephalus. Low incidence of seizures is probably explained by the fact that most of the lesions were sited in the posterior fossa, where the risk of seizure is low; and the supratentorial meningiomas were attached to superior surface of the tentorium, causing displacement of postero-inferior portion of temporal, probably without enough irritation to cause seizures.

Neurological examination revealed 91% of patients had cranial nerve deficit; 50%, ataxia; 38%, cognition deficit; 22%, hemiparesis; 13%, hemianesthesia; and 6%, aphasia. The high incidence of cranial nerve deficits could be attributed to fact that the majority of meningiomas in this series had a large volume at presentation, causing since impairment of vision till hypoacusia in many cases. Unfortunately in Brazil, the diagnosis, mainly in resource-poor areas, is accomplished late, when the patient reaches a reference center.

### Surgical technique

A total of 35 surgical procedures were performed in 30 patients, among whom 3 patients underwent two operations, and another underwent three surgeries. Out of the 3 double twice operations, 1 patient had a combined approach performed in two steps on different days, while the remaining 2 patients had either excessive bleeding or swelling during the initial surgery, which was aborted, partialy removed and continued at a later date to achieve complete removal. Tumor recurrence was the reason for performing three procedures in 1 patient.

Among those patients with supratentorial tumors, 12A tumors were partially removed through an infratentorial supracerebellar corridor, which was complemented 2 months later by an occipital interhemispheric approach. The 2A tumor was removed by a subtemporal approach. The 3 patients in group 3A were operated using an occipital interhemispheric approach, wherein 1 patient needed an external ventricular shunt placement before opening of the dura mater due to obstruction of cerebrospinal fluid (CSF) flow. The 3 patients in group 5A were operated using either a suboccipital approach (1 patient); or a transcortical approach (2 patients) through the second temporal gyrus (T2) when tumors were located more laterally. The 4 patients in group 6A underwent subtemporal (3 patients) or transcortical operations through T2 (1 patient).

**Figure 2:**
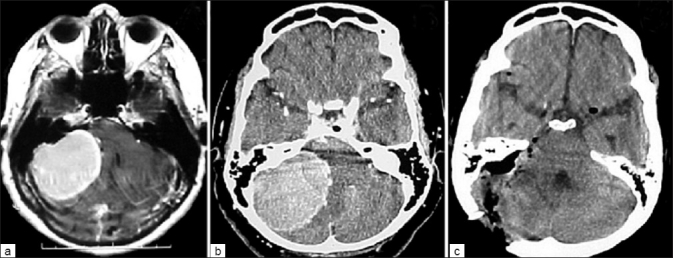
Tumor located at 6B removed by retrosigmoid approach; (a) preoperative MRI (b) preoperative CT scan (c) postoperative CT scan showing total macroscopic removal of the meningioma

Regarding the infratentorial tumors, 10 patients underwent a retrosigmoid suboccipital approach (1 patient with a tumor at 5B position; and 9 patients, at 6B position) [Figure 2]. A combined approach using the infratentorial supracerebellar approach along with bioccipital interhemispheric corridors was employed to treat 3 patients with tumors at 4B position.

**Figure 3:**
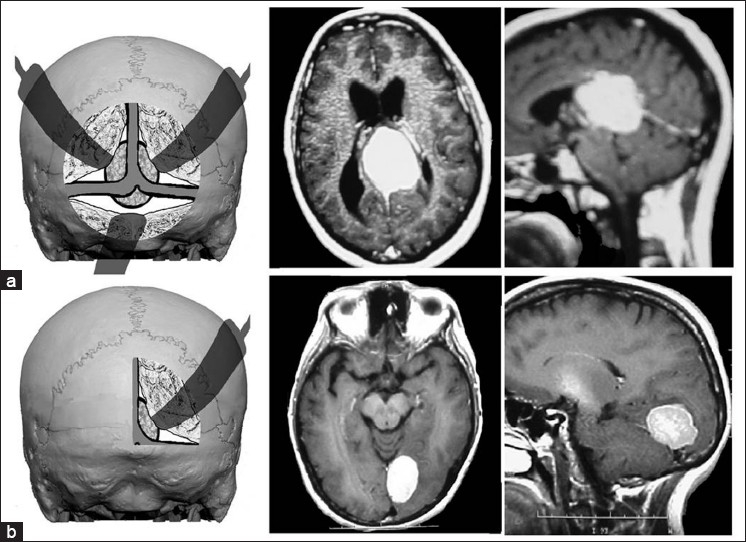
(a) depiction of combined approach exposing supratentorial and infratentorial compartments used to remove tumor located at the 1AB position (patient 3 in Table 1); from left to right on the first line: axial and sagittal views of tumor (b) depiction of suboccipital interhemispheric approach used to remove tumor located at the 3A position (patient 13 in Table 1); from left to right on second line: axial and sagittal views of tumor

Among tumors involving both cerebral compartments, combined approaches were employed on one or two surgical steps in different days. A bioccipital interhemispheric corridor along with an infratentorial supracerebellar approach (exposing both the sagittal and the transverse sinuses in order to use the supratentorial and infratentorial compartments) was used to remove tumors at positions 1AB, 4AB and 45AB [Figure 3]. One of these patients underwent a two-step surgery on different days, 1 week apart. The two other tumors, located at 5AB and 6AB, were removed by combined retrosigmoid and supratentorial (one temporal and other occipital) approaches.

## RESULTS

### Surgical resection

Regular postoperative follow-up included MRI at 6 months and 1 year. Subsequently, periodicity of imaging was based on the initial MRI findings. Simpson’s classification was used to assess the amount of tumor removed.[30] According to this classification, Grade I represents complete removal of the tumor, including resection of underlying bone and associated dura, and has a recurrence rate in 10 years of 9%; Grade II is complete removal plus coagulation of dural attachment with 10-year recurrence rate of 19%; Grade III is complete removal without resection or coagulation of dura, with a 10-year recurrence rate of 29%; Grade IV is subtotal resection and 40% recurrence rate in 10 years. Simpson I resection was achieved in 17 patients; Simpson II, in 11; and Simpson III, in 2 patients. Abnormal thickening of the walls of sinuses seen in the 6-month postoperative MRI was considered residual lesion. Tumors involving both the supratentorial and infratentorial compartments, with involvement of the petrous sinus, and attachment to the torcula (type 4) type prevented Simpson I resection.

Pathology revealed meningioma grade I in 27 patients, according to the World Health Organization classification; grade II, in 2; and grade III, in 1 patient.

### Surgical complications, morbidity and mortality

The transverse sinus was ligated in 3 patients during surgery. Two of these did not develop further neurological deficits, whereas the third patient [Table 1- ID 9] died after ligation of the transverse sinus. She developed venous infarction, severe cerebellar edema, and died on the seventh postoperative day due to pulmonary embolism.

Seven patients evolved presented neurological deterioration, which affected their score on the Karnofsky Performance Scale (KPS). One patient (ID 26) had profuse bleeding during surgery, which needed to be aborted. The patient evolved with severe brain swelling, hemiparesis and incomplete hemianopsia. A decompressive craniectomy was performed, and he was reoperated 2 months later, when a Simpson I resection was achieved. Nevertheless, he scored 70 on the postoperative KPS. Six patients had preoperative hydrocephalus; of them, 2 patients developed shunt dependency and needed ventriculoperitoneal shunt placement (ID 3 and ID 14). We managed the hydrocephalus in 6 patients with external ventricular drainage EVD before surgery. In the postoperative period, we waited for 48 hours with the shunt closed, and if the patient did not present symptom of shunt dependence, we took off the EVD; otherwise, we recommended placement of a ventriculoperitoneal shunt. Among patients with hydrocephalus, 2 (ID 14 and ID 20) patients evolved with mild cognitive impairment and scored 80 on the KPS. One patient (ID 18) had severe brain edema associated with diffuse brain injury during surgery and became dependent at confined to home (KPS score, 50). One patient (ID 22) had intraoperative hypovolemic shock and developed mild cognitive impairment (KPS score, 80). Two other patients evolved with dementia and hemiparesis at the time of tumor diagnosis (KPS score, 80). Their clinical features did not improve significantly after surgery (ID 8 and ID 30).

Seven patients developed postoperative CSF fistula and were treated with external lumbar drainage. One patient (ID 3) developed visual deficit after surgery, when postoperative CT scan revealed a blood clot in the surgical cavity displacing both occipital lobes, which needed to be surgically removed. The patient remained with a small visual field defect without clinical impact (KPS score, 100).

Twenty-two out of 30 patients were able to return to their regular life with no or minimal neurological sequelae, attaining scores of 90 and 100 on the KPS. Two patients scored 70 or less on the KPS after surgery (ID: 18, 26) and were considered as having significant morbidity. Overall, our series revealed 3% mortality and 23% morbidity.

### Follow-up 

Twenty-nine patients had follow-up ranging from 1 to 8 years (average, 4 years 8 months). Two patients had tumor recurrence and died after 6 and 8 years from the first surgery, despite adjuvant therapy. Six patients in our series underwent radiotherapy — 3 patients, due to atypical or malignant pathology; 1, due to imaging signs of tumor regrowth; and 2 patients, due to residual lesion. One patient died after 4 years from a non-tumor-related cause. Clinical evolution has been described above in relation to complications presented.

## DISCUSSION

Tentorial meningiomas are a rare and heterogeneous group of tumors.[9,22] In the period reported on in this study, 454 meningiomas were operated upon at our institution; of these, some 7% were attached to the tentorium. Two other patients were diagnosed with tentorial meningiomas but declined surgical treatment and were followed with serial MRI. Prognosis of tentorial meningiomas depends on position and size of the tumor. Presenting signs and symptoms are usually related to intracranial hypertension due to hydrocephalus or compression of the posterior fossa structures. These include headache, dizziness, gait disturbance and cranial nerve deficits.[13] Our series not only confirmed these findings but also revealed a relatively high incidence of neurological deficits such as hemiparesis and aphasia. This finding may be related to difficulties in accessing neurosurgical treatment at medical centers outside our country. This also explains why 2 patients arrived at our institution already with severe intracranial hypertension requiring urgent surgery.

The classification system for tentorial meningiomas proposed by Yasargil is the most accurate and emphasizes the surgical anatomy.[34] We try to use a simplified classification, dividing patients into six groups, suffixing a letter to denote the location of tumors — whether over or under the tentorium. This was useful to choose the most appropriate approach to the lesion, especially when the mass spread to 2 or more areas. The present classification can strongly give us the visualization as to where the tumor is, without any difficulty or the necessity to watch the image during discussion or to remember where the tumor is; or to determine according to Yasargil classification whether the tumor is T1, T2, T3 and/ or T7. We can say simply denote the location by 4B and we know that the tumor is in torcula and is infratentorial; or 6A, for example, and we know that the tumor is supratentorial and far lateral with respect to the midline. If you observe, the line of possible insertion of meningioma follows a shape of an anchor of ship, hence it is easy for the resident to visualize or imagine where the tumor is when we say 3B, 3A, 5B and so on. This present classification gives us an idea of extension of the tumor; for instance, 3AB represents a meningioma bigger in supratentorial compartment with extension to the infratentorial space. In contrast, a tumor 3BA is a tumor bigger in infratentorial compartment with extension to the supratentorial area.

There are several surgical strategies involved in the treatment of patients with tentorial meningioma, and choosing the most appropriate one is not straightforward.[7,9,15,25,28] In addition to the anatomical data of the individual patient obtained through imaging exams,[10,24] other factors also play an important role in the strategy, such as the clinical condition of the patient and personal experience of the neurosurgeon. The ITSC [25,31] or RS approach was employed in most cases, separately or combined with other craniotomies.[35] Both ITSC and SOIH approaches carry risks while exposing the venous sinuses. On the other hand, when craniotomy is performed, the two approaches provide an intracranial corridor almost free of bridging veins to remove lesions located near the midline. Unfortunately, when the tumor is located at tentorial positions 1 and 3, it is critical to establish the relationship of the lesion with the great vein of Galen and the straight sinus, since both venous structures may be displaced or obstructed.[16,20,23] The RS approach also gives a corridor free of bridging veins; however, when tumor is located near the anterior portion of the petrous bone, the implantation of the tumor may be related to bridging veins from the temporal lobe. Finally, the middle region of the tentorium may encompass sinuses with bridging veins to both infratentorial and supratentorial spaces. One of our patients developed brain engorgement during surgery, compressing the occipital poles against the border of craniotomy. He presented visual and neurological deficits after surgery although the tumor was totally removed through a second approach months later.

The subtemporal or transtemporal approaches were used for tumors located laterally near the petrous bone. The choice between the two approaches was based on the position and caliber of the veins from the temporal lobe draining into the tentorium.[14,24] When surgeons judged that lifting the temporal lobe could cause damage to the veins, a transcortical corridor could be the suitable pathway. Both pathways were able to achieve adequate tumor removal without significant morbidity.

The most vital complications and mortality in our series were related to inadvertent damage to the venous system, affecting 16% of our patients. In this regard, the bridging veins from the cerebellum and temporal lobe, along with the deep veins, deserve special attention as the tentorial meningiomas in the posterior fossa may encompass these structures.[10,29,33] This complication halted two procedures (one had profuse bleeding; and the other, brain swelling — both presumably due to damage of venous drainage); and severely hampered three others, in which the transverse sinus was ligated (one of the patients died). To avoid surgical morbidity due to damage of the venous system, it is necessary to do radiological planning based on angiogram by MRI or digital angiography, and during the surgery, protect and dissect gently the plan interface between the vein and tumor.

Regarding the patient who died after TS obliteration, the sinus was partially obliterated; and after surgical occlusion, an intraoperative edema occurred. Probably the thrombosis extended till the torcula. In the other 2 successful cases with TS ligation, the results could be explained by the hypothesis that there may have been better adaptation of the venous system to chronic subocclusion, The decision to carry out ligation of the transverse sinus was based on the intraoperative view; it means that due to invasion of wall of sinus by the tumor, it was not possible to preserve it and to achieve any anastomosis during that moment, and we had to occlude surgically. Unfortunately, a patient died due to this. In partial occlusion, we avoid, and recommend avoidance of, surgical occlusion.

Four patients had tumors close to the brainstem. However, none had important adhesions or developed damage to the brainstem as reported by others.[4] We also had no cases of postoperative deterioration in cranial nerve function.

The risks of direct surgical approaches for tentorial meningiomas, coupled with high rates of partial resection, have led to increased interest in alternative therapies, particularly radiosurgery. In a recent article by Muthukumar *et al*.[19] based on 41 cases of tentorial meningiomas treated with stereotactic radiosurgery (Gamma knife), tumor reduction was achieved in 18 patients, with no further growth of the tumor in 22 and progression of the tumor in 1 patient (98% control rate), with an average follow-up of 3 years. Radiosurgery is a valuable alternative therapy not only for recurrent tumors but also for some new cases of tentorial meningiomas, especially for small tumors when patients refuse surgery or are not eligible for conventional approaches. In this series, adjuvant treatment was delayed after surgery or recurrence. Six patients underwent radiosurgical treatment. Three initiated radiotherapy after imaging signs of tumor regrowth or residual lesion. During surgery for tumor recurrence, a malignant tumor was disclosed in one of these patients, who died after 96 months. Further, 3 patients (1 with malignant and 2 with atypical meningiomas) needed radiotherapy after the surgery. The patient with malignant tumor died despite adjuvant therapy, after 72 months due to tumor recurrence. However, the 2 patients with atypical tumors, along with the other patient treated for tumor regrowth, are surviving. Certainly, some of our neurological deficits could be avoided with a less aggressive treatment accomplishing subtotal resection with planned adjuvant therapy.

## CONCLUSION

The objective of the meningioma surgery is total removal of the tumor in order to minimize chances of recurrence. However, this objective should not be pursued at all costs, and the decision to leave behind a fragment of the tumor in those cases with adhesions to critical structures can be effective in preserving patients’ neurological status.[4] In our series, a Simpson I resection, which left patients free of tumor recurrence, was achieved in 17 patients, whereas 8 out of 11 patients with Simpson II resection were followed without adjuvant therapy and continue to remain free of active disease after an average period of almost 5 years. The presence of tentorial meningiomas without signs of progressive disease introduces uncertainties over how aggressive resection must be.
